# Female genitalia can evolve more rapidly and divergently than male genitalia

**DOI:** 10.1038/s41467-019-09353-0

**Published:** 2019-03-21

**Authors:** Leigh W. Simmons, John L. Fitzpatrick

**Affiliations:** 1 Centre for Evolutionary Biology, School of Biological Sciences (M092), The University of Western Australia, Crawley, WA 6009 Australia; 2 Department of Zoology/Ethology, Stockholm University, Svante Arrhenius väg 18B, SE-10691 Stockholm, Sweden

## Abstract

Male genitalia exhibit patterns of divergent evolution driven by sexual selection. In contrast, for many taxonomic groups, female genitalia are relatively uniform and their patterns of evolution remain largely unexplored. Here we quantify variation in the shape of female genitalia across onthophagine dung beetles, and use new comparative methods to contrast their rates of divergence with those of male genitalia. As expected, male genital shape has diverged more rapidly than a naturally selected trait, the foretibia. Remarkably, female genital shape has diverged nearly three times as fast as male genital shape. Our results dispel the notion that female genitalia do not show the same patterns of divergent evolution as male genitalia, and suggest that female genitalia are under sexual selection through their role in female choice.

## Abstract (web summary)

Although male genital shape is known to evolve rapidly in response to sexual selection, relatively little is known about the evolution of female genital shape. Here, the authors show that across onthophagine dung beetles, female genital shape has diverged much more rapidly than male genital shape.

## Introduction

Male genitalia are now widely accepted as showing patterns of rapid divergent evolution that are driven by sexual selection^1,2^. However, while there is good evidence that male genitalia exhibit considerable morphological divergence among species^2,3^, the evolutionary rate of that divergence is generally assumed rather than quantified^2^. More importantly, mechanisms of selection responsible for genital divergence, such as species isolation or sexual conflict have been rejected on the grounds that female genitalia do not show the same patterns of rapid divergent evolution as male genitalia^4^. However, research in this field has been strongly biased toward the study of male genital morphology, with little attempt to characterize among-species variation in female genitalia, perhaps because female genital traits are frequently internal and not easily measured^2,5^. For the handful of taxa in which female genitalia have been examined, patterns of coevolutionary divergence in male and female genital traits are emerging^6–10^. These studies call for a greater focus on the study of female genital traits if we are to evaluate proposed mechanisms for the evolutionary diversification of animal genitalia^2,5^. Indeed, recent work with *Drosophila* suggests that natural selection acting on female genital traits might drive the evolution of male genitalia^11^ in much the same way as female reproductive tracts are thought to drive the evolution of sperm morphology^12^.

Female genital traits must interact with male genital traits during reproduction if they are to influence the evolution of male genitalia^13^. Studies of the mechanics of copulation can thus identify potentially informative female genital features that have hitherto gone unnoticed^14–16^. In the dung beetle *Onthophagus taurus*, for example, the male’s aedeagus consists of two segments, the proximal phalobase and the distal parameres. The parameres are inserted into the female’s genital tract where their tips fit into anchorage points, or genital pits, on the internal surface of the pygidium^17^. Multiple mating and post-mating sexual selection are prominent features of the mating system^18,19^ which should promote evolutionary dynamics at the point of genital interaction. Indeed, the shape, but not size, of the aedeagus is under sexual selection, with males possessing a long slender aedeagus being better able to engage the female in copula^20^. Experimental evolution studies have found that the shape of the parameres and the pygidial anchorage points coevolve under sexual selection. Thus, males from populations subjected to sexual selection evolved longer and more slender parameres while females evolved smaller and more internalized genital pits^21^. These studies provide strong evidence that sexual selection is driving the coevolution of interacting male and female genital traits within this species of *Onthophagus*, and point to the onthophagines as an ideal model system with which to examine macroevolutionary patterns of divergence in female genital morphology.

Here we examine the rates of evolutionary divergence in female and male genital shape, and their coevolution across 33 species of onthophagine dung beetles. Sexually selected traits typically exhibit faster rates of phenotypic diversification compared to naturally selected traits^22,23^. The foretibia of dung beetles are adapted for tunneling in soil and dung^24^, so we also examined the rates of divergence in this naturally selected trait for both sexes in order to test the general assumption that genital traits exhibit particular rapid divergence under sexual selection. We find that female genital shape is diverging more rapidly than female foretibia shape, and male genital shape is diverging more rapidly than male foretibia shape. Remarkably, female genital shape is diverging three times faster than male genital shape, potentially driving the correlated evolution of male genitalia.

## Results

### Genital shape

**Fig. 1:**
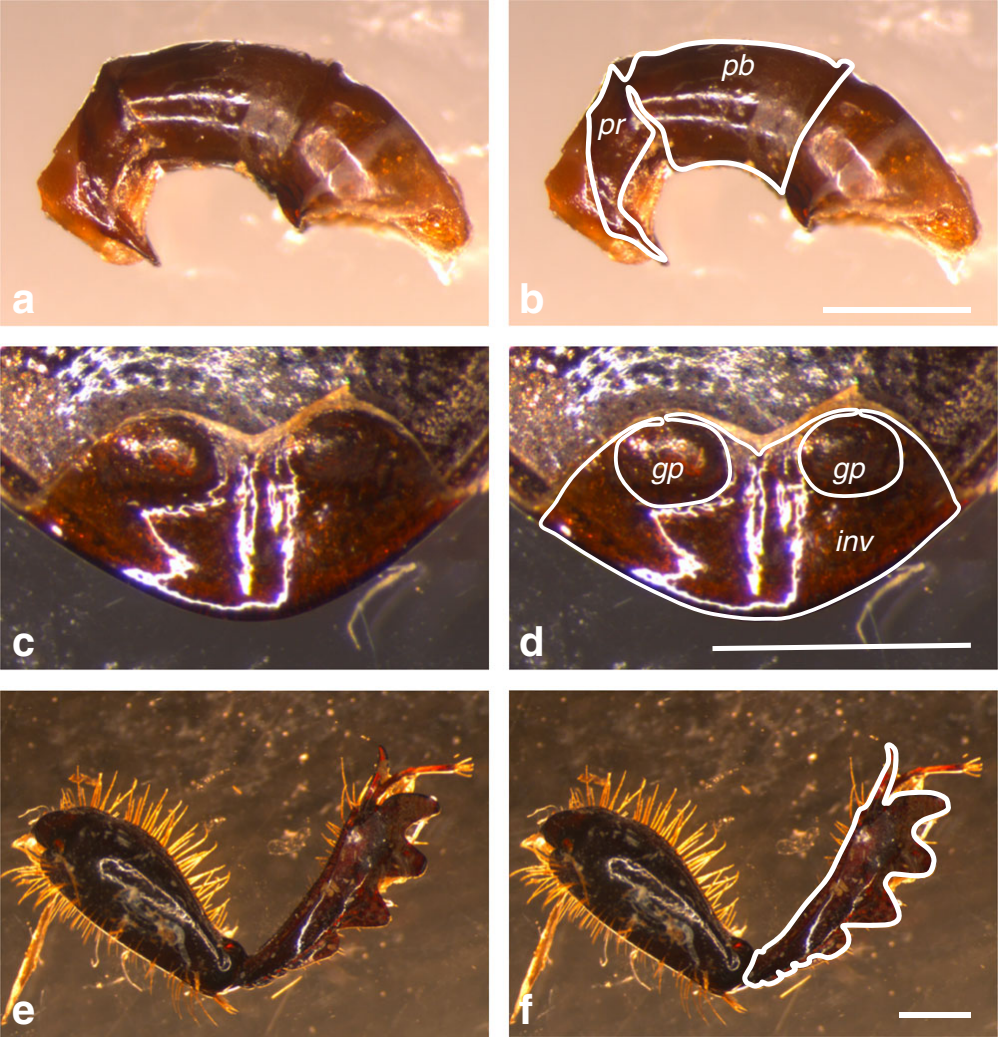
Shape analysis. An example of the application of outlines to the proximal phalobase and distal parameres of the aedeagus (**a**, **b**), the pygidium (**c**, **d**) and the foretibia (**e**, **f**) of *Onthophagus capella*. Scale bar = 1 mm. pr parameres (note the sclerotization of the parameres relative the rest of the distal aedeagus segment), pb phalobase, gp genital pit, inv internal invagination of the pygidium

We used Elliptic Fourier Analysis (EFA) to quantify shape^25,26^. Elliptic Fourier Analysis is an outline-based approach that is particularly well suited to describing complicated and irregular shapes with many concave elements^27^. The method involves describing the outline of a trait using a nonlinear function and comparing variation in the parameters of the fitted functions (the Fourier coefficients) among species and traits^28^. Importantly, it has been used widely to quantify the shape of insect genitalia^29–31^. Twenty harmonics were computed, yielding a multivariate data set of 80 size-normalized coefficients (4 per harmonic) that described the outline of each trait (Fig. 1, and Supplementary Figs. 1–4). To explore whether the number of harmonics chosen might influence our conclusions, we repeated all of our analyses using just ten harmonics. These analyses returned qualitatively similar results (see Supplementary Note 1).

Major axes of shape variation were visualized by subjecting the Fourier coefficients for each trait to separate Principal Component (PC) analyses^25,27^. For pygidium shape, the first two PCs explained 36 and 19% of the variance in pygidium shape respectively; 91% (91%) of the variation in the first (second) principal component was explained by species, and the PC descriptors of pygidium shape were independent of pygidium size (Table 1). We used the application rptR to assess the repeatability of our descriptors of pygidium shape^32^. The among-species repeatabilities for both PCs were significant (PC1: R ± SE = 0.888 ± 0.031; 95%CIs 0.815, 0.932; *P* < 0.001; PC2: R = 0.889 ± 0.030; 95% CIs 0.815, 0.931; *P* < 0.001). The PCs described variation in the presence and shape of genital pits on the internal surface of the pygidium and the depth of its invagination into the female reproductive tract (Supplementary Fig. 2). For aedeagus shape, the first two PCs explained 39 and 24% of the variance in aedeagus shape respectively; 90% (68%) of the variation in the first (second) principal component was explained by species, and the PC descriptors of aedeagus shape were independent of aedeagus size (Table 1). The among-species repeatabilities for both aedeagus PCs were significant (PC1: R = 0.873 ± 0.034; 95%CIs 0.790, 0.921; *P* < 0.001; PC2: *R* = 0.577 ± 0.078; 95%CIs 0.394, 0.709; *P* < 0.001). The PCs described variation in the pointedness of the parameres and their proportional contribution to the aedeagus relative to the phalobase (Supplementary Fig. 3).

**Table 1:** The effect of species and trait size on trait shape

Trait		Effect	F	d*f*	*P* value
Pygidium	PC1	Species	28.62	32, 122	<0.001
		Trait size	0.86	1, 122	0.355
	PC2	Species	39.61	32, 122	<0.001
		Trait size	1.25	1, 122	0.266
Aedeagus	PC1	Species	33.88	32, 114	<0.001
		Trait size	0.01	1, 114	0.930
	PC2	Species	7.29	32, 114	<0.001
		Trait size	0.46	1, 114	0.499
Foretibia	PC1	Species	17.45	32, 224	<0.001
		Sex	70.31	1, 224	<0.001
		Trait size	0.70	1, 224	0.402
	PC2	Species	6.01	32, 224	<0.001
		Sex	34.27	1, 224	<0.001
		Trait size	0.89	1, 224	0.347

Shape is described by the first two major axes of variation (Principle Components) in size-normalized coefficients that describe the outline of each trait

### Fortibia shape

Foretibia shape varied between both species and sex. Again, we visualized shape variation using a principle component analysis. The first two PCs explained 45 and 14% of the variance in foretibia shape respectively. For the first (second) principle component, 74% (54%) of the variation was explained collectively by species and sex (Table 1). The PC descriptors of foretibia shape were independent of foretibia size (PC1: F_1, 224_ = 0.70, *P* = 0.402; PC2: F_1, 224_ = 0.89, *P* = 0.347) and were significantly repeatable (PC1: *R* = 0.619 ± 0.070; 95%CIs 0.455, 0.731; *P* < 0.001; PC2: *R* = 0.384 ± 0.075; 95% CIs 0.227, 0.519; *P* < 0.001). These PCs explained variation in the robustness of the foretibia and in the degree of serration of the outer edge, both among species and between the sexes, with females generally having more robust and serrated foretibia (Supplementary Fig. 4).

### Rates of trait shape evolution

Prior to analyzing shape evolution, we assessed the degree of phylogenetic signal in male and female genitalia and foretibia multivariate shapes to determine if closely related species shared similar traits due to their shared evolutionary history. Male and female genitalia both exhibited significant phylogenetic signal (aedeagus: *K*_mult_ = 0.85, *p* = 0.001; pygidium: *K*_mult_ = 0.78, *p* = 0.001), indicating that closely related species have more similar genital shapes, so that phylogenetic effects significantly explain the evolution of genital shape among onthophagine dung beetles. In contrast, male and female foretibia exhibited lower phylogenetic signals that did not differ statistically from the null model of no phylogenetic signal in the data (male foretibia: *K*_mult_ = 0.51, *p* = 0.11; female foretibia: *K*_mult_ = 0.40, *p* = 0.71).

**Fig. 2:**
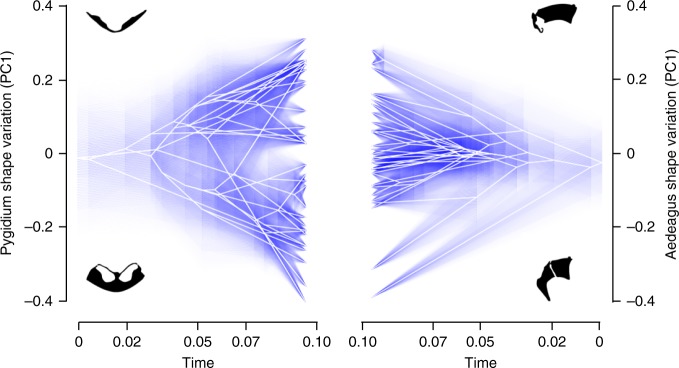
Comparing male and female genital evolution over time. Traitgrams projecting the onthophagine phylogeny into a space defined by pygidium (left) and aedeagus (right) shape variation over time. The y-axis represents descriptions of multivariate genital shape obtained from the first principal component (PC1) of size-normalized Fourier coefficients for female and male genitalia plotted against the relative time from the root of the phylogeny. Traitgrams illustrate greater variation (i.e., more divergence in multivariate shape space) in pygidium shape among closely related species compared to variation in aedeagus shape. Uncertainty around ancestral states over time is depicted such that increasing transparency of the blue lines indicates increasing ancestral state uncertainty. Shape variation is illustrated with outlines from species at opposite ends of multivariate pygydium (*O. aeruginosus* + ve; *O. binodis* -ve) and aedeagus (*O. aeruginosus* + ve; *O. nigriventris* -ve) trait space

To visually compare the evolution of male and female genital shapes over time, we mapped the PC1 scores for male and female genitalia onto the onthophagine phylogeny (Supplementary Fig. 5). As found for male genitalia generally, aedeagus shape is highly variable among species; inspection of the ancestral character estimation of aedeagus shape revealed multiple independent evolutionary shifts towards the extreme ends of the shape distribution in which males have either extended forwardly pointing or stubby backwardly pointing parameres (Supplementary Fig. 5). More significantly, pygidium shape was also highly variable among species, exhibiting numerous independent evolutionary shifts towards the extreme ends of the shape distribution in which genital pits are either present or absent (Supplementary Fig. 5). We visualized the variation in male and female genital shapes over evolutionary time by constructing a traitgram for the aedeagus and pygidium (Fig. 2). Over evolutionary time, the pygidium exhibited significantly more shape variance than the aedeagus (variance ratio test: F_32, 32_ = 2.28, *p* = 0.02), suggesting that evolutionary diversification is more rapid for the pygidium than the aedeagus.

**Fig. 3:**
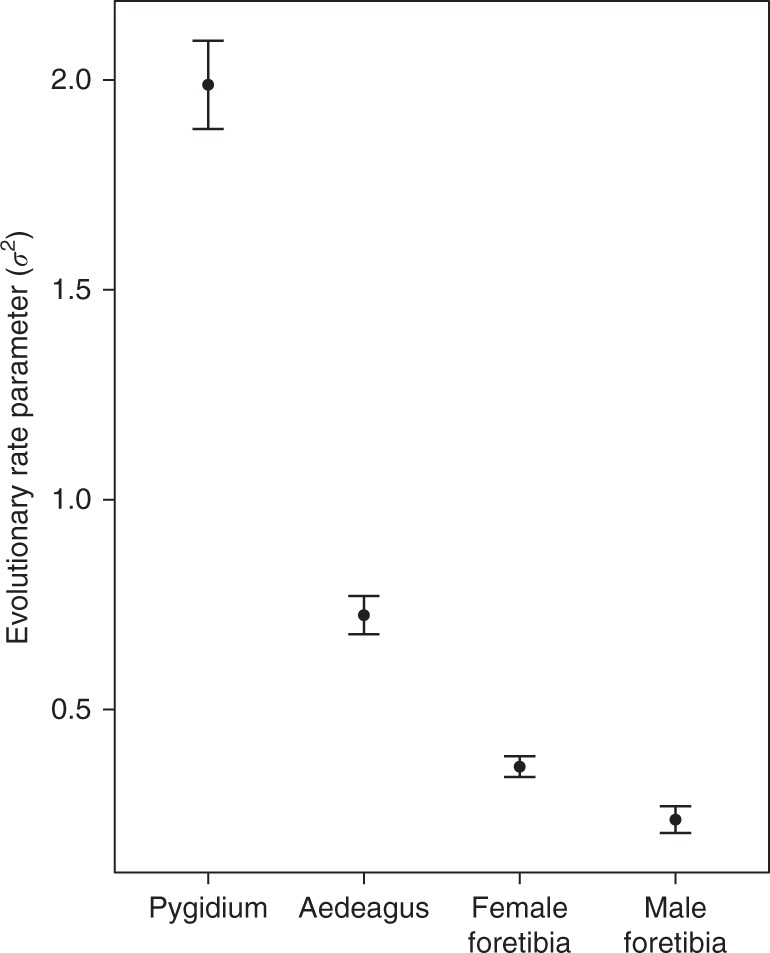
Phylogenetic rate of shape evolution of male and female genitalia and foretibia. Evolutionary rate parameters (*σ*^2^) and their 95% confidence intervals are presented for the pygidium, aedeagus and female and male foretibia. Post hoc pairwise comparisons revealed significant differences in the rate of evolution among all genitalia and foretibia shapes

We calculated and contrasted evolutionary rates of divergence among and between the shapes of male and female genitalia and a non-genital trait, the foretibia (Fig. 1, Supplementary Fig. 4). To do this, we compared the evolutionary rates among genital and foretibia shapes in a single model to determine the relative evolutionary rates of each of these multivariate traits. We detected significant differences in evolutionary rates among male and female genitalia and foretibia shapes (evolutionary rate ratio, *R* = 8.36, *p* = 0.001, Fig. 3). Comparisons of evolutionary rates revealed that the pygidium (evolutionary rate parameter, *σ*^2^_pygidium_ = 1.99) evolved at the fastest evolutionary rate, followed by the aedeagus (*σ*^2^_aedeagus_ = 0.73), female foretibia (*σ*^2^_female foretibia_ = 0.36) and male foretibia (*σ*^2^_male foretibia = _0.24, Fig. 3). Thus, these evolutionary rate parameters revealed that the pygidium evolved 2.7 times faster than the aedeagus, male and female genitalia evolved faster than foretibia (the pygidium evolved 5.5 times faster than the female foretibia, while the aedeagus evolved 3.0 times faster than the male foretibia), and female foretibia evolved 1.5 times faster than male foretibia (Fig. 3). Pairwise post hoc comparisons revealed significant differences in evolutionary rates among all of the shapes assessed (all post hoc pairwise comparisons, *p* = 0.001). We conducted a second analysis in which we considered female and male genitalia alone. This analysis produced qualitatively similar results: female genitalia evolved significantly faster than male genitalia (evolutionary rate ratio, *R* = 2.74, *p* = 0.001; *σ*^2^_pygidium_ = 1.99; *σ*^2^_aedeagus_ = 0.73).

### Correlated evolution in trait shape

**Fig. 4:**
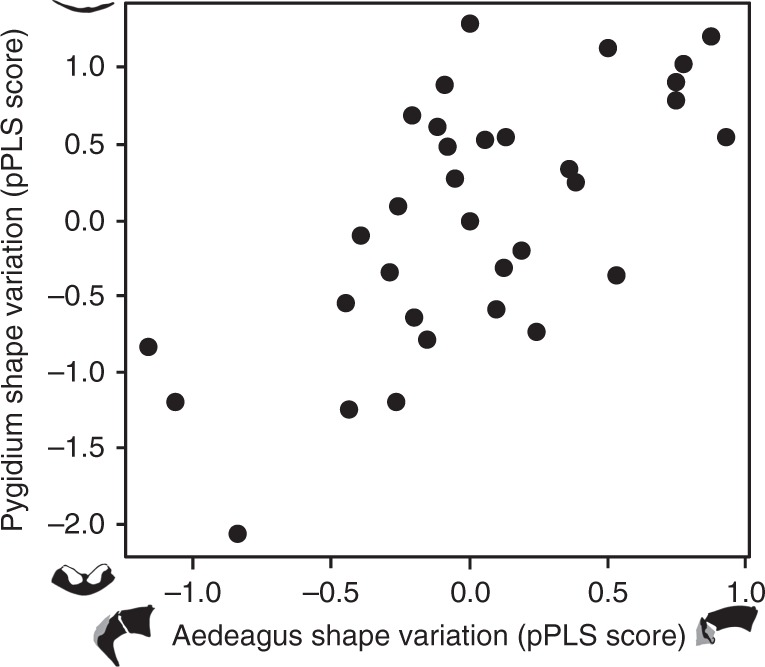
Correlated evolution of female and male genitalia. Plot from phylogenetic two-block partial least squares (pPLS) analyses of female (pygidium) and male (aedeagus) genital shape. Shape variation is illustrated using outlines from species with representative scores along the first latent variable obtained from a phylogenetically controlled partial least-squares analysis of size-normalized Fourier coefficients. These axes correspond closely to the major axes of variation from principal components analyses of shape data prior to controlling for phylogeny (Fig. 2, Supplementary Figs. 2 and 3)

If sexual selection is driving the rapid evolutionary divergence of female genital traits, we would expect to see strong correlations between female genitalia and the male traits with which they interact^2,13^, but no correlation between genital and non-genital traits. Comparing evolutionary correlations between genitalia and foretibia shape among onthophagine dung beetles revealed a strong correlation between the sexes for both genital and foretibia shape, but no evolutionary correlation across genitalia and foretibia shapes. Male and female genital shape were strongly correlated (phylogenetic two-block partial least-squares (pPLS) analyses, pPLS_corr_ = 0.67, *p* = 0.01, Fig. 4), showing that changes in genital shape in one sex are reflected in corresponding changes in genital shape in the other sex. Specifically, in species where the female pygidial invagination is typically shallow and/or with no genital pits, male genitalia have relatively stubby and backwardly pointed parameres. However, in species where females have well defined and deeply set genital pits, male genitalia have forwardly pointed and extended parameres (Fig. 4). Male and female foretibia shape also exhibits strong covariation in multivariate shapes (pPLS_corr_ = 0.80, *p* < 0.001) (Supplementary Fig. 6). In contrast, neither male nor female genital shapes were evolutionarily correlated with male foretibia shape (aedeagus: pPLS_corr_ = 0.53, *p* = 0.12, pygidium: pPLS_corr_ = 0.41, *p* = 0.60) or female foretibia shape (aedeagus: pPLS_corr_ = 0.41, *p* = 0.53, pygidium: pPLS_corr_ = 0.43, *p* = 0.40).

## Discussion

The evolution of male genital morphology has been the subject of considerable research attention, with debates over the roles of species isolating mechanisms, sexual selection via cryptic female choice and sexual conflict remaining unresolved^2,4^. This is perhaps unsurprising, given that most studies of genital evolution have ignored a critical component of mating interactions, the morphology of female genitalia^2,5,13^. Contrary to early views that female genitalia lack the same levels of morphological variation seen in male genitalia^1^, we show that among onthophagine dung beetles, one female genital trait exhibits even greater morphological shape variation than the male genital trait with which it interacts. The pygidium of the female evolved over 5 times faster, and the aedeagus of the male 3 times faster than the foretibia. These accelerated rates of evolution are expected of traits subject to strong sexual selection^22,23^ and confirm the often assumed view that genital traits exhibit rapid divergent evolution. Although male genital shape was found to be coevolving with female genital shape, remarkably, female genital shape appears to be evolving at nearly 3 times the rate of male genital shape.

Theoretical models of female preference evolution predict the establishment of genetic correlations between female and male traits that affect a mating bias^33^. If genital shape is evolving through female choice, interacting female and male genital traits are expected to become genetically correlated^2^ resulting in the concerted evolution of female and male genital shape both within and among species. In *Onthophagus* the aedeagus interacts functionally with the pygidium to affect copulation^17^. Quantitative genetic studies have reported strong genetic correlations between these multivariate female and male genital shapes in *O. taurus*^21^, and manipulation of the strength of sexual selection in this species has documented the correlated evolution of male and female genital traits following just 19 generations of experimental evolution^21^. Moreover, the appendage-patterning genes *homothorax* and *decapentaplegic* are involved in the development of both the pygidium and the aedeagus of *O. taurus* and *O. binodis*, indicating that the same genes have pleiotropic effects on female and male genital shape^34^. The macroevolutionary covariation among species documented here, coupled with the genetic architecture of the aedeagus and pygidium within species of *Onthophagus*, are therefore consistent with a model of genital coevolution involving female choice.

Genital interactions can also be a primary site of sexual conflict leading to sexually antagonistic coevolutionary dynamics between the sexes, where adaptations in genital morphology in one sex impose counteracting selection on genital morphology in the other sex^35^. Sexual conflict can also lead to genetic correlations among traits^35^, and coevolutionary divergence across species^36^. A key distinction between traditional models of female choice and sexually antagonistic coevolution is that in the latter, mating reduces female fitness so that female genital shape should evolve to reduce costly mating^35,36^. Evidence from three species of *Onthophagus* seems more consistent with traditional models of female choice because mating increases offspring survival with no cost to female productivity or lifespan^37–39^. Nevertheless male fitness is strongly affected by number of mates^19,40^ so that female choice is unlikely to be in the male’s best interests, with sexual conflict over mate choice decisions the inevitable consequence^41^. As such, traditional models of female choice and sexual conflict are likely to both contribute to the rapid coevolutionary divergence of female and male genital shape.

Our analysis of foretibia shape was made primarily to provide a contrast between the rates of genital divergence and divergence in a non-genital trait. However, the patterns of foretibia shape variation are interesting in their own right, and deserve some comment. We found considerable among species variation in the robustness of the foretibia and their degree of serration. There was very weak phylogenetic signal in foretibia shape, indicating considerable evolutionary lability. The onthophagines are tunneling beetles that provision broods underground, and they use their foretibia to tunnel through the soil and to cut fragments of dung that are carried underground and packed into brood balls. Recent work with *O. taurus* and *O. illyricus* has revealed that both inter- and intra-specific divergence in foretibia morphology is paralleled by variation in nesting depth; populations with the deepest brood burial depth were found to have broader foretibia^24^. These findings suggest that the considerable evolutionary divergence in foretibia shape found across the onthophagine phylogeny may be explained in part by variation in ecological and environmental variables such as soil type or moisture content, and brood burial behavior. We found a pattern of strong coevolutionary divergence between female and male foretibia shape. This is perhaps unsurprising, given they are homologous traits and that both males and females use their foretibia for tunneling. Interestingly, however, we also found sexual dimorphism in foretibia shape, with females having the more robust and serrated foretibia. During brood provisioning it is the female who is primarily responsible for cutting fragments of dung and packing them into the brood ball. In *O. taurus*, males can sometimes contribute to brood provisioning, although when they do, females allocate 84% of their time to collecting and packaging dung while males allocate only 43% of their time^42^. Moreover, males will only assist in provisioning when there is no risk of competition from rival males^43^. We might expect therefore that selection via brood provisioning might act more strongly on the foretibia of females. Indeed, we found that female foretibia shape is diverging 1.5 times faster than male foretibia shape, contributing to sexual dimorphism in this naturally selected trait.

In conclusion, our comparative analysis of onthophagine dung beetles has revealed that female genital morphology is highly variable among species, and that female genital shape is diverging more rapidly than a naturally selected trait and nearly three times as rapidly as male genital shape. The collective evidence from within and among species studies of these beetles suggests that female genitalia are a female choice trait that imposes selection on male genital morphology. More broadly, our analyses provide novel empirical insight into the evolutionary dynamics of female preference evolution. Theoretical models of preference evolution have converged on the expectation that changes in female preferences will drag male sexual traits with them^33^. However, current models of preference evolution fail to consider predictions about rates and patterns of radiations in male traits and female preferences^33^. Numerous studies have found that male traits subject to female preference are evolving more rapidly than naturally selected traits^22,44–46^. If female preference traits drive the evolution of male sexual traits^11,12^ we might expect preference traits to evolve more rapidly. To our knowledge ours is the first to show that female preference traits, in this case female genital morphology, show faster rates of evolutionary divergence than the male traits on which they act. Future theoretical studies need to consider how rates of female preference evolution should vary among different models of preference evolution, while empirical studies should examine the rates of female preference evolution for genital and non-genital ornaments in order to determine whether female preferences generally undergo more rapid evolutionary divergence than male sexual traits, or whether this pattern is unique to the genital morphology of onthophagine beetles.

## Methods

### Experimental material

We characterized the size and shape of the aedeagus (male genitalia, *n*_total_ = 148), pygidium (female genitalia, *n*_total_ = 157), and male (*n*_total_ = 128) and female foretibia (*n*_total_ = 131) from 33 species of *Onthophagus* dung beetles, with a mean ± SE of 4.9 ± 0.2 male and 4.8 ± 0.2 female individuals per species (Supplementary Table 1). All beetles were collected from natural populations and had been lodged in university or museum collections. Ethical approvals or collection permits were not required for this research. Sample size for each species was determined a priori by the number of individual beetles that were made available to us from collection curators, with the exception of *O. taurus* and *O. binodis* that were available locally. The sample sizes for these species were set to the mean sample size across all other species. Foretibia shape could not be quantified on all individuals because they were sometimes found to be damaged. Each trait was quantified independently, and blind to other trait values. Because we had variation in the number of samples available for each species and trait type, we conducted sensitivity analyses to confirm that sample size did not affect the conclusions drawn from our analyses (Supplementary Note 1).

### Shape analysis

Each structure was dissected and mounted on a glass slide with the aid of a smear of petroleum jelly. An image was captured using a Leica MZ6 trinocular microscope fitted with a Leica DFC 290 camera. Outlines could not be digitized directly from the images because the traits of interest could not be removed from, or in the case of the pygidium were integral to, associated body structures. Thus, for the aedeagus (Fig. 1a), an outline was drawn around the structures of interest, the heavily sclerotized regions of the proximal (phalobase) and distal (parameres) (Fig. 1b). For the pygidium (Fig. 1c), an outline was drawn around the invagination of the exocuticle, delineating articulation points or genital pits where present (Fig. 1d). The right foreleg of males and females were similarly mounted for image capture (Fig. 1e) and an outline drawn around the tibia, including the tibial spur (Fig. 1f). Care was taken to orient the structures from each specimen in the same orientation and plane. Outlines were then digitized using tpsDig^47^ and Elliptical Fourier Analysis performed in EFAWin^47^. Twenty harmonics were sufficient to reconstruct the original shapes (Supplementary Fig. 1), and their coefficients were normalized for size using the methods of Kuhl & Giardina^48^. This method is effective provided that size-shape relationships are isometric. To test for the efficacy of size-normalization, the area delineated by the outline of each trait was measured using ImageJ and included in analyses to confirm that trait shape was independent of trait size.

### Phylogenetic analyses

To account for evolutionary relationships among species we employed a molecular phylogeny constructed with Bayesian likelihood methods using four nuclear and three mitochondrial genes of 33 species of onthophagine dung beetles^23^. All analyses were based on the 2D-matrix of size-normalized Fourier coefficients and implemented using the package *geomorph* v3.0.1^49^ in R v3.3.0 (R Development Core Team, 2016). The *geomorph* package is typically used to assess multidimensional trait data generated using landmark-based shape data (i.e., requiring an *n* × *p* × *k* input matrix). However, like landmark-based geometric morphometric data, EFA coefficients are multidimensional traits that describe the outline of a shape and are readily incorporated into downstream analyses in *geomorph* in the absence of defined landmarks (D.C. Adams, personal communication). Specifically, analyses were performed by treating the 2D matrix (i.e., an *n* × *p* matrix, rather than the traditionally used 3D array that assumes landmark-based shape data) as the dependent variable. Because we were interested in comparing rates of evolution of different shape components, we determined the mean shape for each shape component (e.g., aedeagus, pygidium, male foretibia and female foretibia) for each species prior to analyses.

### Assessing phylogenetic signal

Prior to analyzing shape evolution, we assessed the degree of phylogenetic signal in male and female genitalia and foretibia to determine if closely related species shared similar traits due to their shared evolutionary history. Due to the high-dimensionality of the shape data included in our analyses we used Adams’^50^ *K*_mult_ statistic, a multivariate generalization of Blomberg et al.’s^51^ *K* statistic, to assess phylogenetic signal. Analyses were implemented using the *physignal* function in *geomorph*. Increasing values of *K*_mult_ indicate increasing phylogenetic signal in the data set. Specifically, *K*_mult_ = 0 suggests no phylogenetic signal in the data, while if shape variation is influenced by the phylogeny under a Brownian motion process then *K*_mult_ = 1. Statistical significance of *K*_mult_ was determined by comparing the observed values with values obtained by using 1000 randomized trait values across the phylogeny^50^. Significant *K*_mult_ values indicate that multivariate traits evolve differently from the null model of no phylogenetic signal (i.e., *K*_mult_ > 0).

### Comparing genital shape evolution over time

We visualized the evolution of male and female genital shapes over time using two complimentary approaches. In these analyses we extracted the first Principal Component (PC1) from size-normalized Fourier coefficients for male and female genitalia using sex-specific Principal Component Analyses to describe the complex multivariate shape variation in male and female genitalia separately (Supplementary Figs. 2 & 3). First, we used the *contMap* function in the R package *phytools*^52^ to reconstruct continuous trait evolution in a maximum likelihood framework for both male and female genital shape (Supplementary Fig. 5). We used the visualization of male and female genital shape evolution to highlight the appearance of covariance in male and female genital shape, which we then test formally (see below), and to identify species where male and female genital shape shifted to the extreme ends of the shape distribution. Second, we used the *fancyTree* function in *phytools* to create a traitgram projecting the phylogenetic tree into male and female genital shape space^52,53^. We then used an F test to compared variances between male and female genital shapes (note that as a phylogenetically corrected variance ratio test does not currently exist, this analysis did not control for phylogenetic effects).

### Assessing evolutionary correlations among multivariate traits

We evaluated whether multivariate traits were evolutionarily correlated using phylogenetic two-block partial least-squares (pPLS) analyses implemented with the *phylo.integration* function in *geomorph*^54^. We evaluated the covariance between all possible combinations of male and female genitalia and foretibia shapes. Observed covariance values from pPLS analyses are statistically compared to a null distribution generated using 10,000 phylogenetic permutations, where data from one multivariate trait is randomly shuffled among tips on the phylogeny and assessed relative to the other multivariate trait.

### Comparing rates of multivariate shape evolution

We compared the rates of multivariate trait evolution among genitalia and foretibia using the *compare.multi.evol.rates* function in *geomorph*^55^. This approach derives evolutionary rate parameters of multivariate traits (*σ*^2^, the multivariate rate of change for each trait) by determining the morphospace distance between species following phylogenetic transformation. To compare evolutionary rate parameters (*σ*^2^) among multivariate traits, the rate of shape variation for each trait is calculated and then used to calculate an evolutionary rate ratio (*R*) among multivariate traits. To determine if the evolutionary rate ratio differs significantly between or among multivariate traits, the observed rate is compared to a null evolutionary rate matrix derived from a simulation, using 1,000 phylogenetic permutations, and a common rate matrix for all traits. To deal with our input data, we concatenated the 2D matrices from the shape components included in the analyses. In all analyses we specified that multivariate traits came from different subsets (i.e., specified the argument Subset = False) to allow for comparisons among multivariate shapes describing distinct phenotypic components^55^. From a practical point of view, specifying that shapes are derived from different structures alters the way the evolutionary rates are calculated by dividing the assembled vector of Euclidian distances from the origin for each species by the number of species analyzed. Consequently, the rates analyses assess how shape variation for each shape component changes in multivariate space from the mean multivariate trait shape for all species included in the analyses, thereby providing a measure of shape divergence that is normalized for each shape component. After estimating evolutionary rates for each multivariate shape, we estimated 95% confidence intervals around *σ*^2^ values for each shape by bootstrapping (1000 iterations) data from individuals used to calculate species shape means.

We compared evolutionary rates of multivariate traits using a two-step analytical approach. First, we compared the evolutionary rates of male and female genitalia and foretibia shapes in a single analysis to directly contrast the evolution of these multivariate traits. Since this analysis compared four multivariate traits (i.e., aedeagus, pygidium, male foretibia and female foretibia) we report the global evolutionary rate ratio *R* for the analysis, the evolutionary rate parameter *σ*^2^ for each multivariate trait, and the post hoc pairwise comparison between groups using the pairwise. *p* value object in *compare.multi.evol.rates*. Second, since only male and female genitalia exhibited significant phylogenetic signal consistent with a Brownian motion model of multivariate trait evolution, we performed an additional analysis comparing the evolutionary rates between male and female genitalia alone.

### Reporting Summary

Further information on experimental design is available in the Nature Research Reporting Summary linked to this article.

## Supplementary information

Supplementary Information

Reporting Summary

## Data Availability

The R code to replicate the analyses is available at the University of Western Australia Research Repository, 10.26182/5becb699400fe (ref. ^56^).

## Data availability

All data that underlie the results are available at the University of Western Australia Research Repository, 10.26182/5becb699400fe (ref. ^56^).
