# Supplementary material for: Female genitalia can evolve more rapidly and divergently than male genitalia
Source: Nat Commun. 2019 Mar 21;10:1312. doi: 10.1038/s41467-019-09353-0 (PMC6428859; doi:10.1038/s41467-019-09353-0)
Supplement: Supplementary file 1 — Supplementary Information [file 41467_2019_9353_MOESM1_ESM.pdf]

SUPPLEMENTARY INFORMATION

Female genitalia can evolve more rapidly and divergently than male genitalia

Simmons & Fitzpatrick

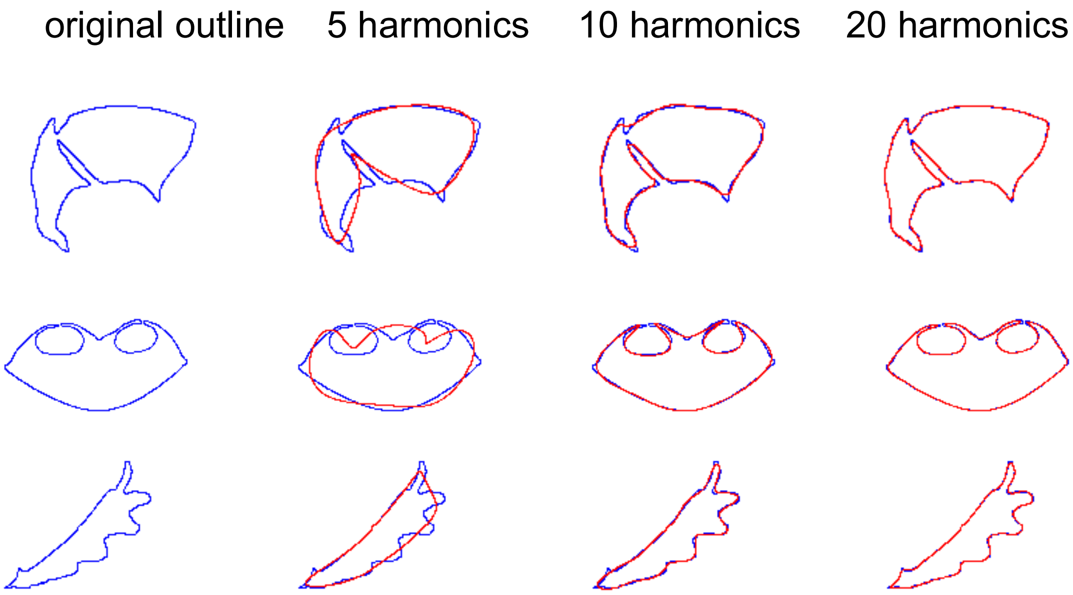

Supplementary Fig. 1. Elliptical Fourier analysis of shape variation. Inverse representation of the elliptical Fourier series (red) superimposed on the original outlines (blue) for the aedeagus (top), pygidium (middle) and foretibia (bottom) of *Onthophagus capella*. Inverse representations are shown for increasing numbers of harmonics.

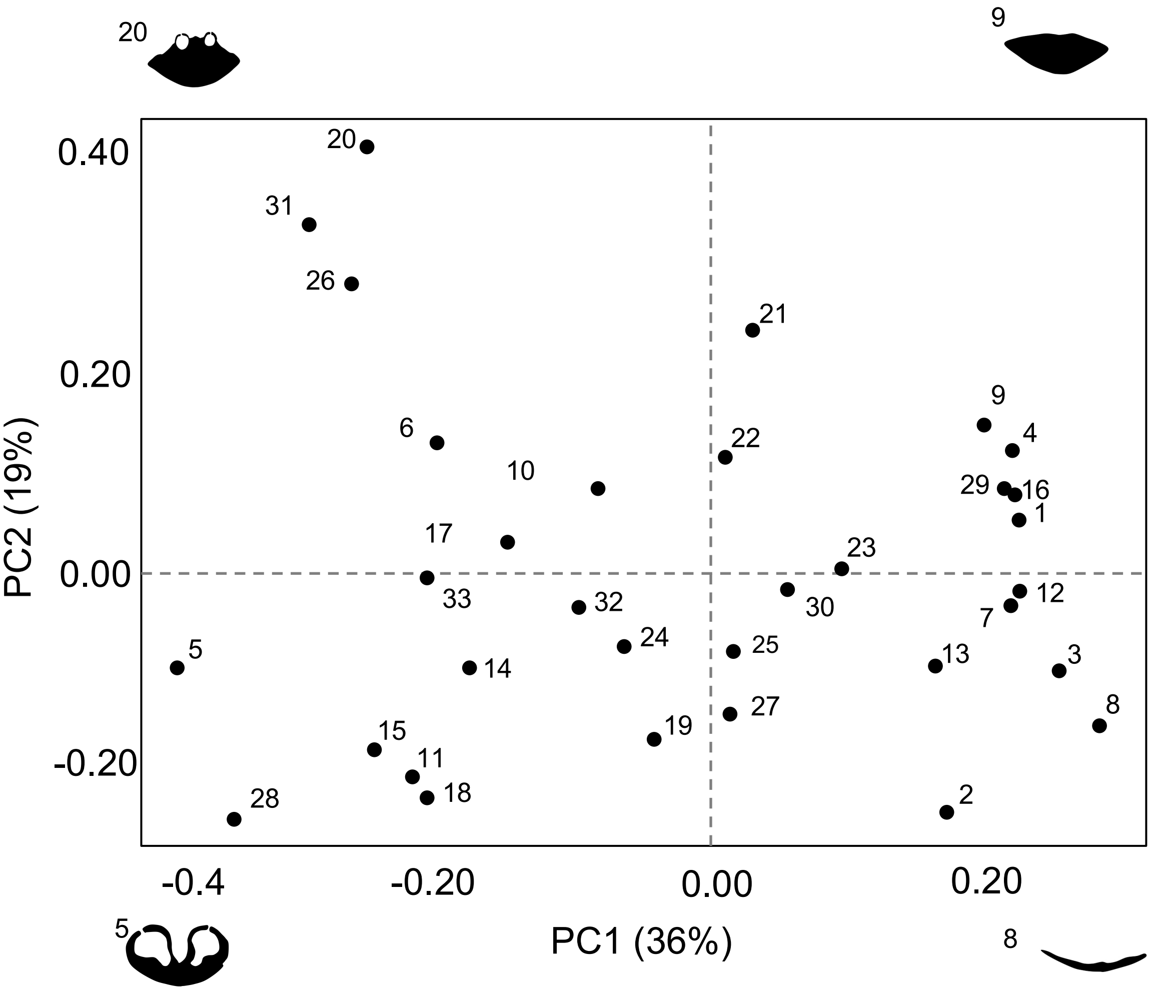

Supplementary Fig. 2. Among species variation in the shape of female genitalia. Shape is described by the first two axes from a principal components analysis of the Fourier coefficients describing the outline of the invagination of the pygidium. Points depict the species mean coordinates on PC1 and PC2. One species silhouette is shown for each end of the distributions in PC1 and PC2 trait space. Numbers provide species identities given in Supplementary Table 1.

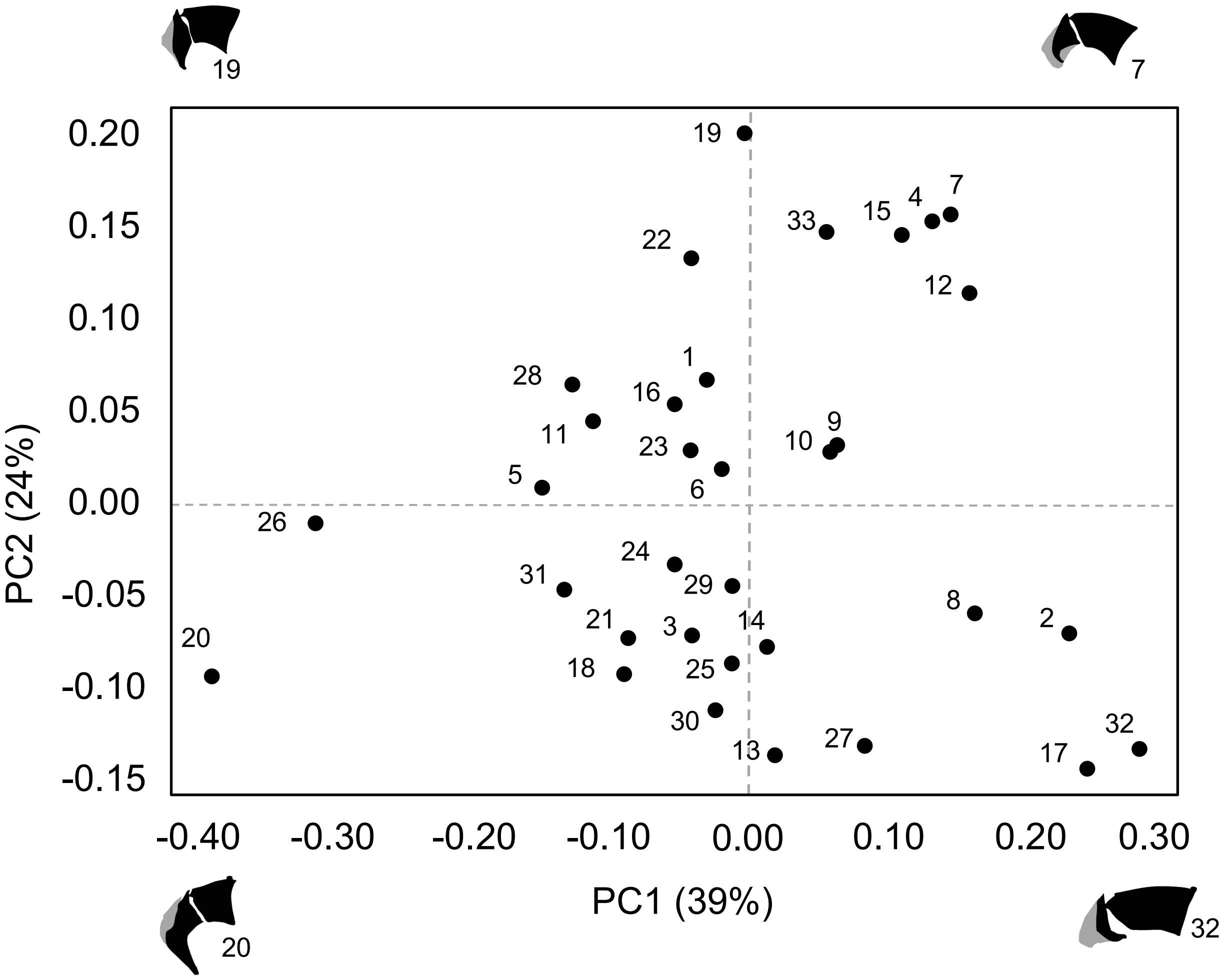

Supplementary Fig. 3. Among species variation in the shape of male genitalia. Shape is described by the first two axes from a principal components analysis of the elliptic Fourier coefficients describing the outline of the aedeagus. Points depict the species mean coordinates on PC1 and PC2. One species silhouette is shown for each end of the distributions in PC1 and PC2 trait space. The gray area for each example shows the soft tissue that was not included in the outline analysis (see Fig. 1). Numbers provide species identities given in Supplementary Table 1.

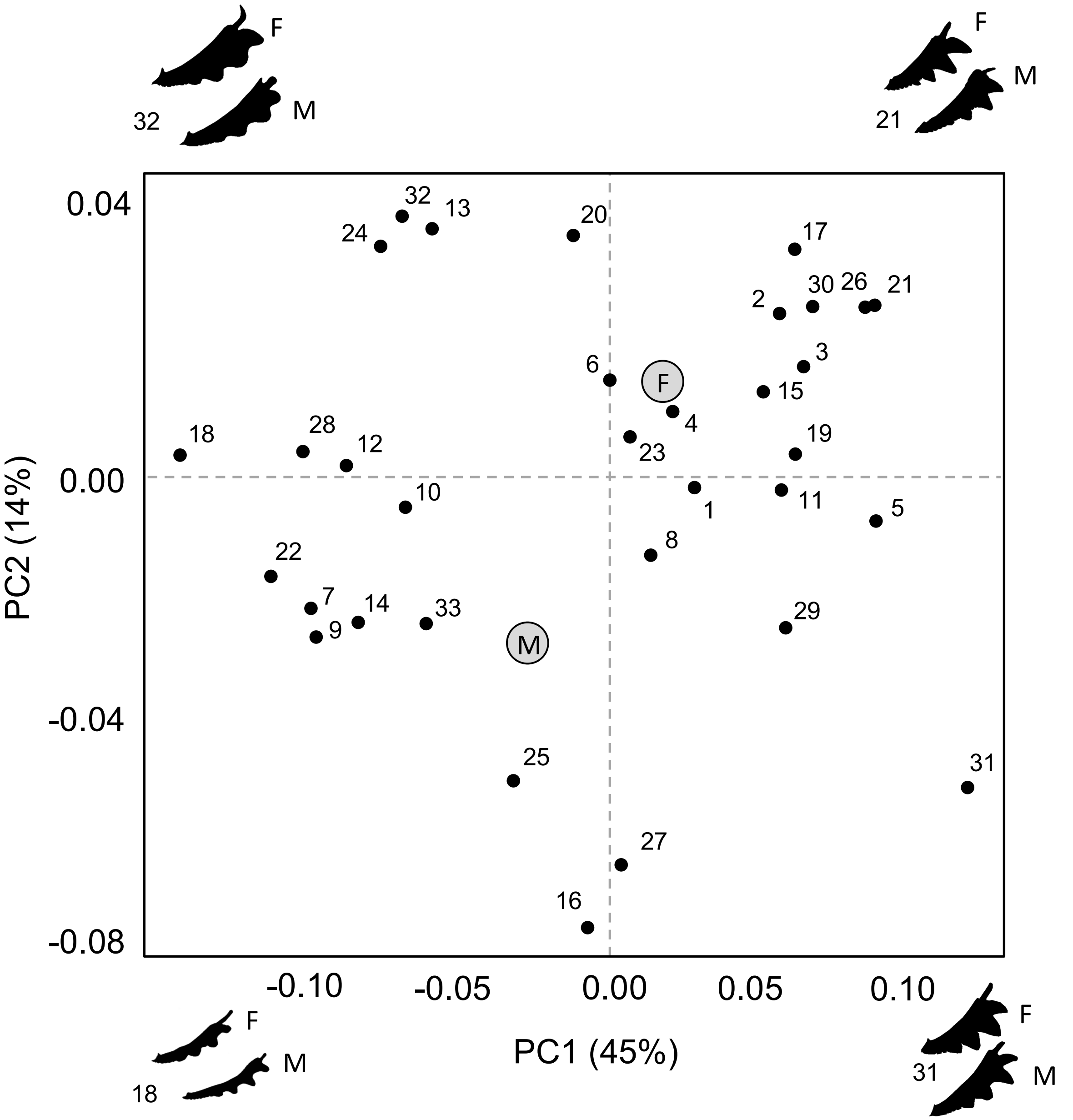

Supplementary Fig. 4. Among species variation in the shape of the foretibia. Shape is described by the first two axes from a principal components analysis of the elliptic Fourier coefficients describing the outline of the foretibia. Points depict the species mean coordinates on PC1 and PC2. The mean male (M) and female (F) coordinates on PC1 and PC2 are shown as large grey circles. One species silhouette is shown for species at each end of the distributions in PC1 and PC2 trait space. Numbers provide species identities given in Supplementary Table 1.

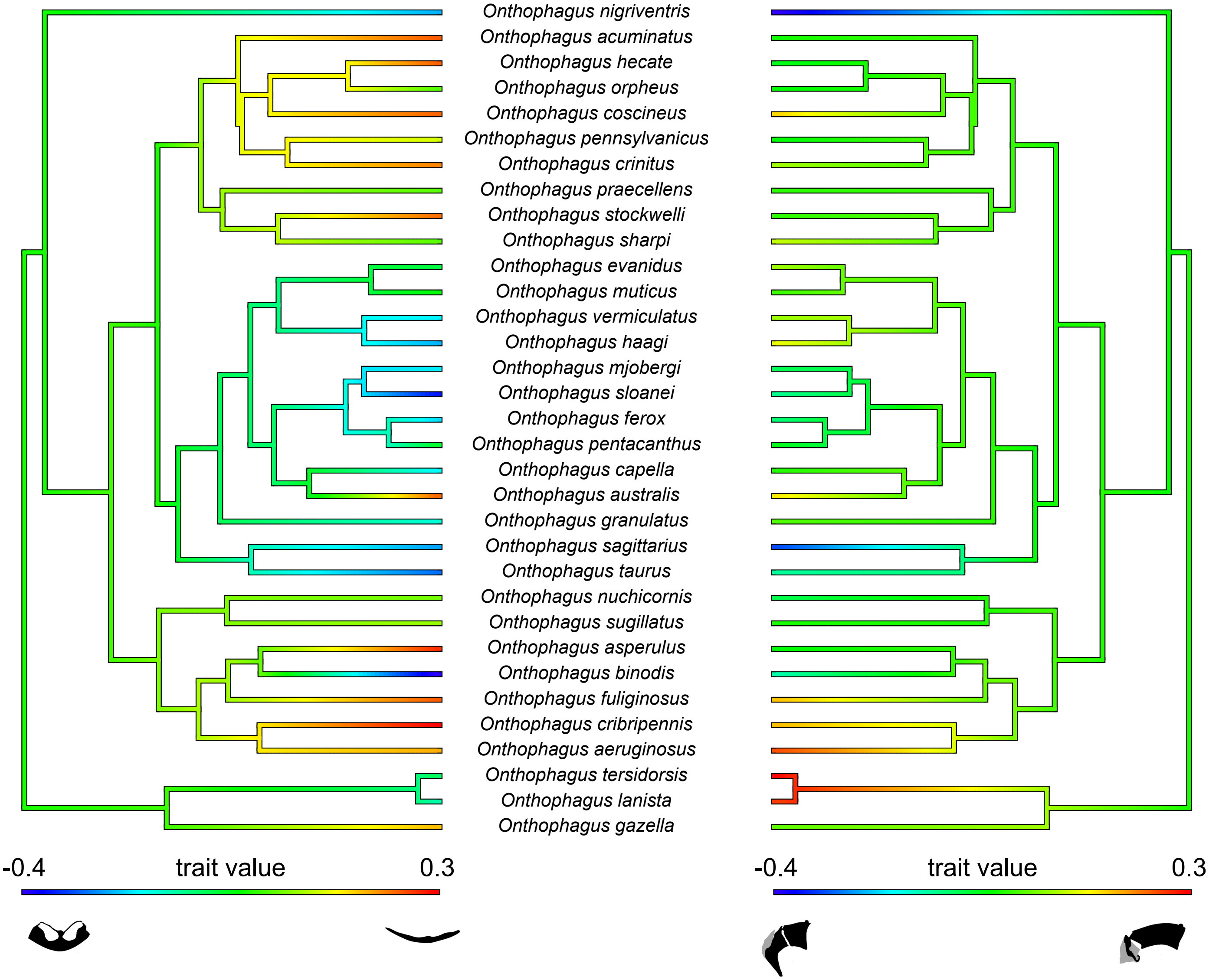

Supplementary Fig. 5. Ancestral character estimate of pygidium (left) and aedeagus (right) shape variation among onthophagine dung beetles. Shape is described using the first principal component scores obtained from size-normalized Fourier coefficients for female and male genitalia. Colors plotted on the phylogenies represent the observed and reconstructed values of genital shape. For the pygidium, blue colors correspond to species with genital pits that are deep in the female body cavity, green colors correspond to species with genital pits that are closer to the genital opening, and red colors correspond to species with no genital pits (Supplementary Fig. 2).For the aedeagus, blue colors correspond to species with extended and forwardly pointed parameres, green colors correspond to species with short backwardly pointing parameres, and red colors correspond to species with greatly reduced parameres (Supplementary Fig. 3).

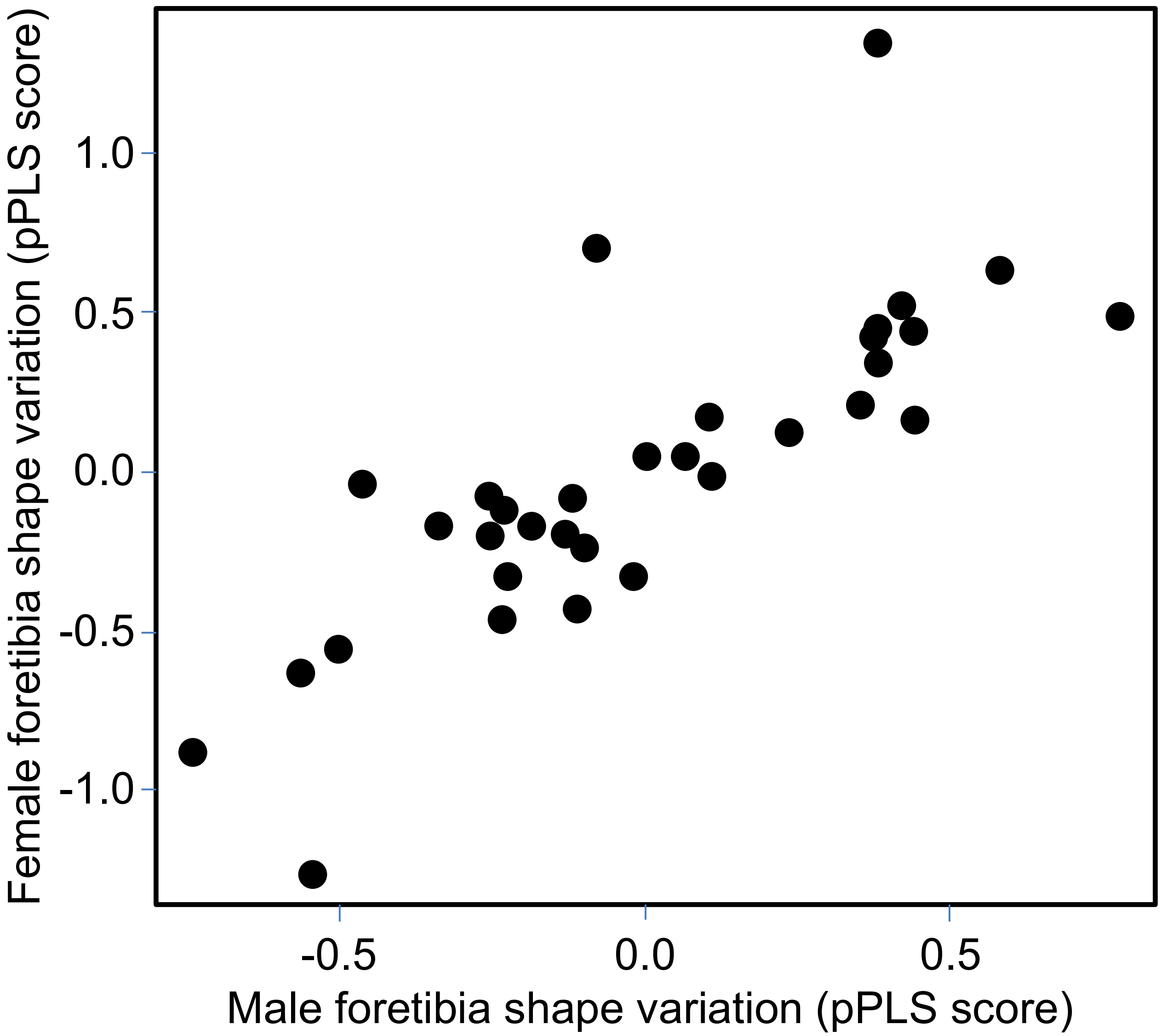

Supplementary Fig. 6. Correlated evolution of female and male foretibia. Plot from a phylogenetic two-block partial least squares (pPLS) analyses of female and male foretibia shape. These axes correspond closely to the major axes of variation from principal components analyses of shape data prior to controlling for phylogeny (Supplementary Fig. 4).

Supplementary Table 1. Sample sizes for shape analyses of the 33 species of *Onthophagus* dung beetles examined in the study.

		Sample size			
	**Species**	**Aedeagus**	**Pygidium**	**Male legs**	**Female legs**
1	*O. acuminatus*	5	6	5	3
2	*O. aeruginosus*	2	2	4	4
3	*O. asperulus*	6	6	1	4
4	*O. australis*	4	5	4	4
5	*O. binodis*	4	5	4	5
6	*O. capella*	3	1	3	1
7	*O. coscineus*	4	5	2	2
8	*O. cribripennis*	3	5	3	4
9	*O. crinitus*	3	4	5	2
10	*O. evanidus*	5	5	2	5
11	*O. ferox*	5	6	5	5
12	*O. fuliginosus*	5	5	4	4
13	*O. gazella*	4	5	3	4
14	*O. granulatus*	5	6	5	4
15	*O. haagi*	5	4	5	5
16	*O. hecate*	5	6	5	5
17	*O. lanista*	6	6	5	5
18	*O. mjobergi*	3	3	5	5
19	*O. muticus*	3	3	3	3
20	*O. nigriventris*	6	5	5	4
21	*O. nuchicornis*	5	5	3	5
22	*O. orpheus*	3	2	3	2
23	*O. pennsylvanicus*	4	4	5	4
24	*O. pentacanthus*	6	5	5	4
25	*O. praecellens*	5	6	4	4
26	*O. sagittarius*	4	6	5	5
27	*O. sharpi*	5	5	4	3
28	*O. sloanei*	3	4	3	3
29	*O. stockwelli*	5	5	4	5
30	*O. sugillatus*	6	7	5	5
31	*O. taurus*	3	5	4	4
32	*O. tersidorsis*	6	6	2	5
33	*O. vermiculatus*	7	4	3	4

Supplementary Note 1: Sensitivity analyses

Sampling effort. We had considerable variation in the number of samples for each species that were available. Our analyses did not directly account for differences in sample size among species. However, as all downstream analyses were based on species mean shapes, differences in sample sizes among species would only influence the results if there were substantial within-species variance in shapes. Such within-species variance in shapes would be particularly problematic if it were greater than the between-species variance, in violation of a common assumption in comparative analyses. We have demonstrated that this is not the case: shapes were repeatable among species and there was greater between than within-species variance in shape (see main text). Nevertheless, we were surprised to find little discussion in the literature about how different sampling effort among species can influence how much variance is explained by Fourier coefficients. We used two approaches to determine whether sampling effort might influence our results, which yielded qualitatively similar results to the analyses presented in the main text.

First, we randomly selected a single individual from each species to include in our analyses. In effect, we equalized the sampling effort to an n=1 for each species, such that the total number of individuals matched the total number of species (n=33 species). Using this dramatically reduced within-species sampling dataset, our results were not qualitatively altered. As in the analyses presented in the main text, male and female genitalia shapes exhibited significant phylogenetic signal (aedeagus: K_{mult} = 0.69, p = 0.001; pygidium: K_{mult} = 0.61, p = 0.005), male and female foretibia exhibited low phylogenetic signal (male foretibia: K_{mult} = 0.50, p = 0.10; female foretibia: K_{mult} = 0.40, p = 0.70), female genital shape evolved faster than male genital shape, which in turn evolved faster than male and female foretibia shape (evolutionary rate ratio: R = 9.88, p = 0.001; evolutionary rate parameter: $\sigma^2_{pygidium}$ = 3.04, $\sigma^2_{aedeagus}$ = 1.16, $\sigma^2_{female\ foretibia}$ = 0.47, $\sigma^2_{male\ foretibia}$ = 0.31), and finally, male and female genital shapes were correlated ($pPLS_{corr}$ = 0.69, p = 0.005) and male and female foretibia shapes were correlated ($pPLS_{corr}$ = 0.64, p = 0.05).

Second, we performed the same set of analyses on a subset of species in our dataset where at least five individuals were sampled. In this second set of analyses, we equalized the sampling effort to five for each species (i.e. if more than five individuals were sampled we randomly selected five individuals to represent the species in the analyses). This inclusion criterion reduced our dataset to roughly half of the original number of species (n=16, although note that we only considered female and male genital shapes in these analyses due to differences in sample sizes among shapes making it impossible to assess a more complete dataset given the inclusion criteria we applied here). Despite this reduction in the number of species assessed, we once again found qualitatively similar results to our full analyses presented in the main text. Specifically, male and female genital shapes were correlated ($pPLS_{corr}$ = 0.82, p = 0.05) and female genital shape evolved faster than male genital shape (R = 2.31, p = 0.001, $\sigma^2_{aedeagus}$ = 1.48, $\sigma^2_{aedeagus}$ = 0.64). Together these analyses suggest that variance in within-species sampling effort does not qualitatively alter our results.

Number of harmonics. In the main text we used 20 harmonics to characterize trait shapes. This yielded 80 parameters for each individual in the analysis. To ensure that our conclusions were not affected by the number of parameters, we repeated all analyses using just 10 harmonics, or 40 parameters. The results were qualitatively similar. As in the analyses presented in the main text, trait shapes varied significantly among species but were independent of trait size (Supplementary Table 2). Male and female genital shapes exhibited significant phylogenetic signal (aedeagus: $K_{mult} = 0.86$, p = 0.001; pygidium: $K_{mult} = 0.79$, p = 0.001), male and female foretibia exhibited low phylogenetic signal (male foretibia: $K_{mult} = 0.52$, p = 0.12; female foretibia: $K_{mult} = 0.41$, p = 0.68), female genital shape evolved faster than male genital shape, which in turn evolved faster than male and female foretibia shape (evolutionary rate ratio: R = 8.79, p = 0.001; evolutionary rate parameter: $\sigma^2_{aedeagus} = 1.96$, $\sigma^2_{aedeagus} = 0.71$, $\sigma^2_{female\ foretibia} = 0.34$, $\sigma^2_{male\ foretibia} = 0.22$), and finally, male and female genital shapes were correlated ($pPLS_{corr} = 0.65$, p = 0.02) and male and female foretibia shapes were correlated ($pPLS_{corr} = 0.80$, p = 0.001). Thus, our conclusions were not altered by the number of harmonics chosen to describe trait shape.

Supplementary Table 2. The effect of species and trait size on the first two major axes of variation (Principle Components) in size-normalized coefficients that describe the outline of genital traits and the foretibia of onthophagine dung beetles. In this analysis 10 harmonics were used in the elliptical fourier analysis.

Trait		Effect	F	df	*P*
Pygidium	PC1	Species	28.54	32, 122	<0.001
		Trait size	0.87	1, 122	0.354
	PC2	Species	42.33	32, 122	<0.001
		Trait size	1.25	1, 122	0.266
Aedeagus	PC1	Species	34.13	32, 114	<0.001
		Trait size	0.01	1, 114	0.936
	PC2	Species	7.21	32, 114	<0.001
		Trait size	0.46	1, 114	0.499
Foretibia	PC1	Species	17.39	32, 224	<0.001
		Sex	69.51	1, 224	<0.001
		Trait size	0.82	1, 224	0.367
	PC2	Species	6.04	32, 224	<0.001
		Sex	35.49	1, 224	<0.001
		Trait size	0.74	1, 224	0.391
